# Annual Meeting of the Medical Society of the County of Erie

**Published:** 1880-02

**Authors:** 


### ANNUAL MEETING OF THE MEDICAL SOCIETY OF THE COUNTY OF ERIE.

*Stated Meeting, January 13, 1880.*

THE PRESIDENT, DR. SYLVESTER F. MIXER, IN THE CHAIR.

Present—Dr. S. F. Mixer, President; Dr. D. W. Harrington, Secretary; and Doctors White, Rochester, Miner, Boardman, John Cronyn, Gay, Thomas Lothrop, L. P. Dayton, B. L. Lothrop, O'Brien, Abbott, Howe, Phelps, Mynter, Slacer, Samo, Nichell, Keene, McPherson, Elwood, Hoyer, Earl, Folwell,

Dorland, Moody, Brecht, Sloan, Banta, Davidson, E. E. Storck, Campbell, Dambach, Dagenais, King, Briggs, Lynde, Hauenstein, Daggett, Barker, Bailey, Lapp, Nott, Hopkins, Diehl, Hartwig, Haberstro, Packwood, Sonnick, Schade, Bartlett, Kamerling, Brooks, Wyckoff, Macniel, Wetzel, Hill, Thurber, Jansen.

Drs. C. A. Wall, Jos. W. Keene, M. Hartwig, L. L. Banta, and Dr. Bideman were elected members.

Drs. W. W. Turner and Julius Krug were proposed for membership and referred to Committee on Membership.

The Special Committee, of which Dr. J. F. Miner is chairman, appointed to confer with The Charity Organization Society, presented their report.

The librarian report was referred to a committee, composed of Drs. Howe, Folwell and Thos. Lothrop.

Prof. White submitted the following preambles and resolution and moved that copies of the same be sent to the members of the Legislature from this District:

Whereas, The Erie County Medical Society fully recognize that medical science is in a great measure indebted to experimental physiology for its existence and development; and

Whereas, It apprehends that certain articles recently published in some of the metropolitan journals, foreshadow injudicious action on the part of certain so called philanthropists, which, if successful, will, in the opinion of this Society, prove highly detrimental to the progress of medicine as a science and its advance as an art; therefore,

*Resolved*, That our delegates to the State Society be and they are hereby instructed to vigilantly guard against and to vigorously oppose the adoption by the Legislature of any measures which may tend to limit or rescind the privileges of employing living animals for experimental purposes, which privilege is now granted to those engaged in physiological investigations.

The report and the motion were adopted.

Prof. White moved the adoption of the following preambles and resolutions and also that a copy of the same, signed by the officers of the Society, be sent to Governor Cornell through Prof. Miner:

Whereas, The Health Officer of the Port of New York is soon to be named by Governor Cornell; and

Whereas, The important office is for the entire State, and therefore his selection should not be confined to any section or locality; and

Whereas, The selection for this important position has for many years been made exclusively from the eastern portion of the State; therefore, be it

*Resolved*, That in the opinion of this Society this appointment is now due, and therefore should be awarded to the western portion of the State.

*Resolved*, That this Society, representing the medical profession of the County of Erie and city of Buffalo, heartily commend to His Excellency Governor Cornell for nomination to this important office, Prof. Julius F. Miner, M. D.

*Resolved*, That Prof. Miner having been all his life a steadfast Republican, having achieved success as a general practitioner, as a teacher of surgery, and as an editor, and being well known throughout the State as a distinguished member of the profession, we believe him eminently well qualified for the discharge of the duties devolving upon the Health Officer and earnestly request his appointment.

*Resolved*, That we solicit this appointment in the conviction that it will be acceptable to the great body of the medical profession and eminently satisfactory to all interested in the commerce of the port of New York.

The preambles and resolutions, and the motion relating to them, were unanimously adopted.

The following officers were elected for the ensuing year:

President, Dr. F. F. Hoyer; Vice-President, Dr. John Hauenstein; Secretary, Dr. D. W. Harrington; Librarian, Dr. J. B. Samo; Treasurer, Dr. W. C. Phelps; Board of Censors, Drs. Nichell, Hoyer, J. C. Green, Sloan and Briggs.

On motion of Dr. Howe the President and Secretary were authorized to fill vacancies, should they occur among the delegates to the meeting of the State Society.

The retiring President read his address on "Sanitary Science," which was an able discussion of this important subject in its reference to sanitary matters in Buffalo, and the agencies to which they are entrusted.

The meeting then adjourned.

